# Fractures in General Practice

**Published:** 1908-10-24

**Authors:** 


### THE GENERAL PRACTITIONER'S COLUMN.

[Contributions to this Column are invited, and if accepted will be paid for.]

### FRACTURES IN GENERAL PRACTICE.

The fact that we may be unexpectedly called upon to deal with any form of fracture invests the treatment of these injuries with special interest. The alarm and pain caused the patient, the anxiety of his friends, their demand for an immediate diagnosis, and often prognosis, the visible results of failure or success, the infinite variety, often tax the capabilities of the most experienced. Perfect results, always expected, nay, demanded, by patient and friends, and often promised by the surgeon, are not always obtainable.

Do not let a long list of favourable cases make us forget that sooner or later, in spite of all possible care, tedious and unsatisfactory ones will occur. Thus fractures near to or into joints, fractures and dislocations combined, fractures near the elbow in youth, high and obscure shoulder injuries, and of the hip in the aged are always to be treated with respect.

Nearly forty years of military, colliery, quarry, factory, and union work have presented me with what drapers call a large and well-selected assortment. Take the following contrasted cases:—(1) A gentleman broke his leg, tibia about four inches, fibula about five above the ankle. The surgeon who saw him first applied splints, and brought him home comfortably. The patient somehow laboured under the impression that he would soon be about again—nor did it look likely to give more than ordinary trouble at first sight. I was thankful, however, afterwards that I had been much more guarded in my prognosis; partly owing to a long and severe bout of cramp, to which he was especially subject, a very large amount of blood was poured into and around the joint. Bullæ formed, and for some days the case assumed a most threatening aspect. It was six months before the patient walked again, and even then he was a little lame from joint stiffness.

(2) An old man over seventy years of age was knocked down between a motor and a butcher's cart; he had the ordinary Pott's fracture, but was very rheumatic, and I thought he would be disabled for some time. His leg was lightly bound to a comfortable pillow and diligently bathed with spirit lotion; no splints were used, and he was at work again in less than five weeks. The agent and surgeon to an insurance company offered him a considerable sum, which, on my advice, he accepted.

(3) A surgeon, on being presented with a fracture and dislocation of the elbow in a child of seven, remarked: "It will be a tedious business, and never quite like the other, but it will be a useful joint." The child was taken away and other advice obtained; some criticism was said to have been expressed or implied, and a much more favourable opinion given. Result to the second surgeon—a very long, anxious, and unprofitable case; ultimate deformity, with poor movement.

(4) As a result of an injury in youth, a man about thirty had ankylosis of the left knee at an awkward angle. He then broke his thigh rather high up; the thigh muscles were wasted, while those of the hip were much developed. As the case presented unusual difficulties, a consultation with a well-known surgeon was asked for, who suggested the continuance of the double inclined plane for a rather longer spell than usual, but was far from sanguine as to a good union. On his second visit he thought an operation would be necessary, and in the meantime left the attendant surgeon to his own devices. The patient's health deteriorated under the confinement, for he was naturally an impatient man, though an intelligent one. I advised a stretcher with canvas laced down the middle to permit of the necessary attention, and also suspended over a cross-bar at the knees. Extension was easy, the bent position comfortable, and he was moved about where he wished. The handles of the stretcher at either end were placed over the first or second bar at head or foot of bed for change and attention; the knee bar was not fixed, but suspended from the horizontal for comfort's sake. The whole thing was a sort of hammock and stretcher combined, but divided down the centre. This arrangement answered admirably, and the bone united perfectly. I have frequently used this simple device for many years past, and always with comfort to the patient and satisfaction to myself. The position is so natural that there is little attempt to vary it. Natural wants are easily managed by raising either end of the stretcher; the bed can be kept perfectly clean and dry, or re-made single-handed. The hammock is padded by a folded blanket down the back as far as the buttocks, and by a smaller one under each leg and thigh.

Recently a little girl with old tubercular disease of the left knee and right ankle broke her right thigh. She is now walking about, the thigh united, and both the knee and ankle much better for six weeks' rest. I know of no other method which would have given anything like such good results, for the little patient never had a bad night from first to last. No splinting, or as little as possible, has been my motto for very many years; I have always blamed long and unnecessary confinement for most of the tedious, muscle-wasted, joint-set chronic cases. I could quote hundreds of instances to prove how little restraint is really required. Fortunately of late there has been a vast improvement in this respect in general practice.

